# Edinburgh Royal Infirmary—Treatment of Acute Rheumatism

**Published:** 1894-03-24

**Authors:** 


### EDINBURGH ROYAL INFIRMARY.

### Treatment of Acute Rheumatism.

The treatment of this common and painful affection naturally divides itself into the general management of the case and special treatment directed towards the relief of the more severe symptoms which may arise in its course.

(1) *General Management.*—In perhaps no disease is the part of the treatment included under this head more important than in rheumatic fever, in view of the serious complications, such as peri- and endo-carditis, which may to a great extent be averted or modified by it. In patients of known rheumatic tendency an incipient attack, often ushered in by a characteristic sore throat, may be prevented by speedy confinement to bed and the use of salicin compounds.

In all cases it is essential that the patient should be confined to bed, the sheets being removed as tending to chill the body on becoming soaked with the copious perspiration. He should also be clad in flannel for a like reason, and this should be insisted on even in those who dislike woollen material in contact with the skin. The patient should be warmly, though not too warmly, covered with blankets, and must be warned against the danger of exposure to cold. Though the temperature in ordinary cases does not range high, food should be confined to fluids, with the addition of plain pudding.

As regards drugs salicin and its derivatives are the substances most in favour in cases of acute rheumatism, and may said to exert a special influence on the disease. The compound most in favour here is the salicylate of sodium given usually in dozes of ten grains every two hours to start with, and regulating the amount by the relief of pain and reduction of the temperature, or it may be given till it produces the characteristic singing in the ears. It should, however, be discontinued if much sweating ensues, or if there be any signs of cardiac depression.

The greatest care must be taken not to expose the patient to cold during examination of the chest, though frequent auscultation is desirable in order to be apprised at the earliest moment of any cardiac complication.

In protracted cases the long duration of the disease is frequently due to anæmia, and speedy improvement follows the administration of iron, arsenic, and cod-liver oil. The patient also should not be permitted to leave his bed till the temperature has kept at normal for at least a week, and the absence of complication ascertained by careful physical examination. The chief symptoms requiring special treatment are cardiac complication, hyperpyrexia, and pain in the joints.

(2) *Cardiac Complications.*—In both pericarditis and endocarditis occurring in the course of rheumatic fever, the chief factor in the treatment is securing for the patient the most absolute rest. All other measures are secondary to this, and active treatment in these affections is no longer the rule. Poultices and fomentations to the precordial region are the agents chiefly employed, though cold in the form of ice bag and blisters have also their advocates. These diseases require separate description, but their frequency in connection with rheumatism makes it necessary to mention them, and it should not be forgotten that in children pericarditis may appear among the earliest symptoms of an attack.

(3) *Hyperpyrexia.*—Though the temperature does not range high (101 deg. to 102 deg. Fahrenheit) in ordinary cases, and calls for no special measures for its reduction, it occasionally happens that it runs to 108 deg. Fahrenheit and upwards, this forming a complication at once sudden and dangerous in the extreme if not promptly met by appropriate treatment. This is the condition occasionally spoken of as "cerebral rheumatism." Should the temperature rise to 105 deg. Fahrenheit, a bath cooled with ice must be at once prepared, as if it registers 106 deg. Fahrenheit before the patient can be placed in it fatal results are to be feared. So rapid is this rise that the thermometer placed in the rectum may continue to rise while the patient is in the bath, but soon the cold water abstracts the heat and a fall begins, which must be allowed to continue till a point midway between the original temperature and normal has been reached, when the patient is removed rapidly and put to bed, when it continues to fall for some time.

(4) *Relief of Pain.*—The joint pains in acute rheumatism form one of the most characteristic symptoms of the disease, but are often so severe as to make the slightest movement agony to the patient, and call for special treatment. In all well marked cases the affected joints should be enveloped in wool and bandaged, and relief may be obtained by the use of hot alkaline fomentations—clothes saturated in a strong hot solution of bicarbonate of soda. In more severe cases a useful application is obtained thus: ℞. Chloral hydrate; camphor, ad ʒii.; water to ℥iii., which may be applied on lint. When a single large joint, such as the knee, is severely affected, and is greatly distressing the patient out of proportion to the general severity of the attack, a most useful application is a blister, four inches long by an inch broad, applied transversely round the limb above the joint. In rare cases of long-standing pain a certain amount of atrophic change takes place in the surrounding muscles, and the joint assumes a position of flexion for which extension by weight and pulley is required.

